# Supplementary material for: Tango-seq: overlaying transcriptomics on connectomics to identify neurons downstream of Drosophila clock neurons
Source: bioRxiv. 2024 May 22:2024.05.22.595372. Preprint. [Version 1] doi: 10.1101/2024.05.22.595372 (PMC11142192; doi:10.1101/2024.05.22.595372)
Supplement: Supplement 1 [file NIHPP2024.05.22.595372v1-supplement-1.pdf]

Figure S1: Timecourse of expression of the postsynaptic nuclear tdTomato reporter with *Pdf-GS* used to express the *trans*-Tango ligand.

A: *Pdf-GS > trans-Tango; UAS-mGFP, QUAS-tdTomato-NLS* flies were raised on standard food and then switched to food with 500 μg/mL mifepristone added and dissected after a 1, 3 or 7 days, or switched to fly food with 500 μg/mL EtOH (vehicle) for the no mifepristone sample. Antibodies to GFP (cyan) label the LNvs and antibodies to RFP (magenta) label postsynaptic targets. Antibodies to Vri (yellow) label the nuclei of clock neurons. Brains were dissected and fixed at ZT14 when Vri levels are high.

B: *Pdf-GS > trans-Tango; UAS-mGFP, QUAS-tdTomato-NLS* flies were raised on food with 500 μg/mL mifepristone so that *Pdf-GS* is active throughout development. Adult flies were dissected at ZT14 on the first day after hatching from the pupal case, and stained as above.

Figure S2: *trans*-Tango using two different DN1p lines shows sparse synaptic connectivity to other clock neuron groups.

A: Maximum projections of *CNMa-Gal4* > *UAS-myr-GFP* (left) and *DN1-Gal4* > *UAS-myr-GFP* (right). GFP (cyan) and Vri (yellow) are shown for each line. The *CNMa-Gal4* labels 6 neurons per hemisphere and the *DN1p-Gal4* labels 10-12 DN1ps per hemisphere.

B. Confocal z-stack of fly brains from *DN1p-Gal4* > *UAS-myr-GFP, trans-Tango; QUAS-tdTomato-NLS* (top) *and CNMa-Gal4* > *UAS-myr-GFP, trans-Tango; QUAS-tdTomato-NLS* (bottom). Representative antibody staining for GFP (cyan), tdTomato (magenta), and Vri (yellow) are shown as well as merged images (right).

C. Graphs quantify the number (top) and proportion (bottom) of tdTomato-labeled neurons from each clock cell type (N=10 hemispheres for *CNMa-Gal4* and N=5 hemispheres for *DN1p-Gal4*). Bar plots display mean value with error bars indicating SEM.

Figure S3: Biological sequencing replicates integrate well and produce a high-quality dataset for post-integration analysis.

A: Graphs show violin plots of the number of individual genes identified (nFeatures), number of individual sequencing reads mapped to a transcript (nCount), and the percentage of reads from mitochondrial genes (% mt) before (left) and after trimming (right) the dataset for each cell separated for biological replicates 1 (red) and 2 (blue).

B: Uniform manifold approximation and projection (UMAP) plot showing Canonical correlation analysis (CCA) based integration of the two biological replicates with individual cells color-coded by replicate as in A.

C: Volcano plot shows genes with high variability between cells in the integrated dataset. Significant variability is a factor of overall expression level of a gene and a z-score derived from the dispersion (a function of variance). The top 20 most variable genes are labeled.

D: Violin plots showing the nFeatures, nCount, and % mt for individual clusters derived from the integrated data set. The consistency in these features between clusters indicate they are unlikely to be driving cluster assignment.

Figure S4: s-LNv projections are very close to γ lobe Kenyon cells.

A reconstruction from the hemibrain (v1.2.1) viewer showing the 4 s-LNvs from the right hemisphere (blue), the outline of the right mushroom body calyx (grey), and a selection of γ lobe Kenyon cells (pink). The s-LNv projections pass over the calyx and into the cell body layer of the MB, close to γ lobe Kenyon cells.

62. Zheng, G.X., Terry, J.M., Belgrader, P., Ryvkin, P., Bent, Z.W., Wilson, R., Ziraldo, S.B., Wheeler, T.D., McDermott, G.P., Zhu, J., et al. (2017). Massively parallel digital transcriptional profiling of single cells. Nat Commun *8*, 14049. 10.1038/ncomms14049.

A

Pdf-Geneswitch > TT; tdTomato-NLS
Raised Mif -

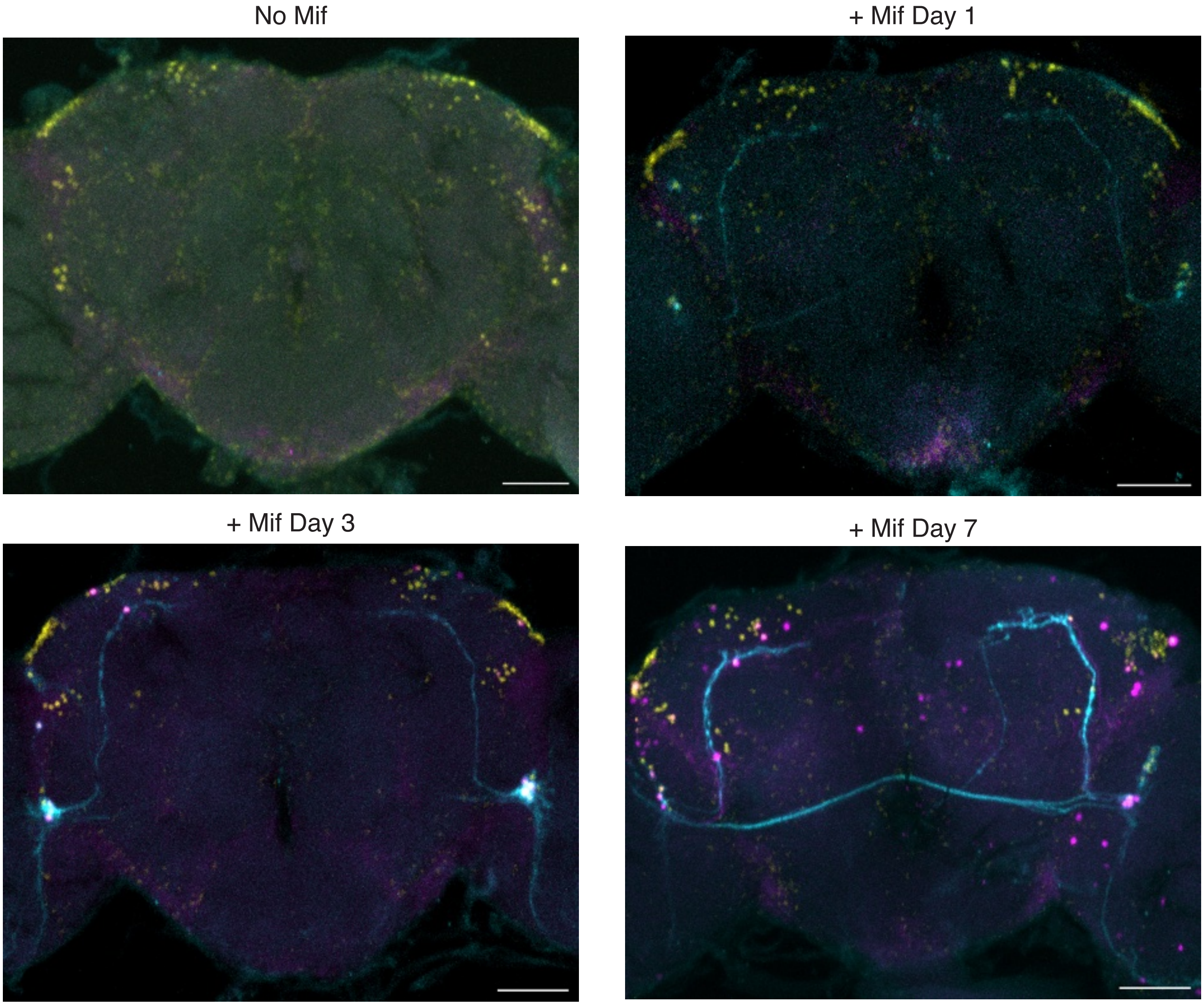

B

Pdf-Geneswitch > TT; tdTomato-NLS
Raised Mif + < 1 day post-eclosion

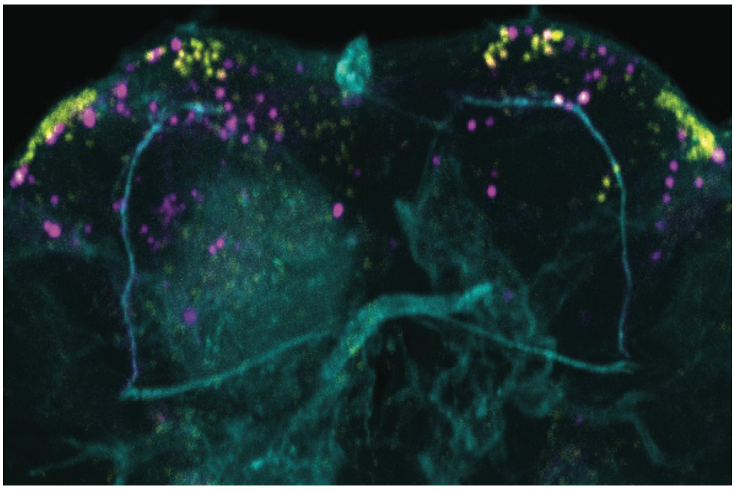

Fig. S1
Ehrlich et al.

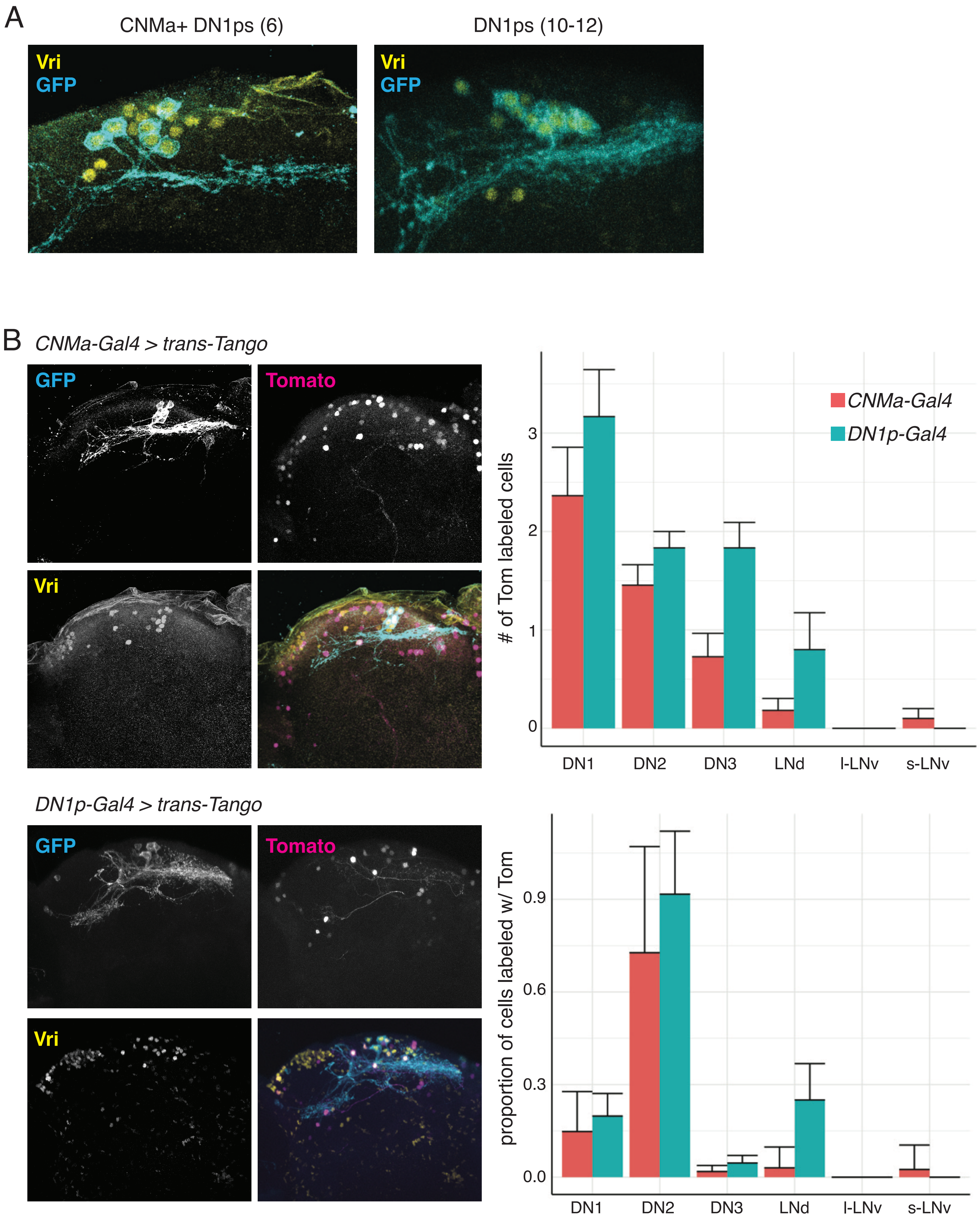

Fig. S2, Ehrlich et al.

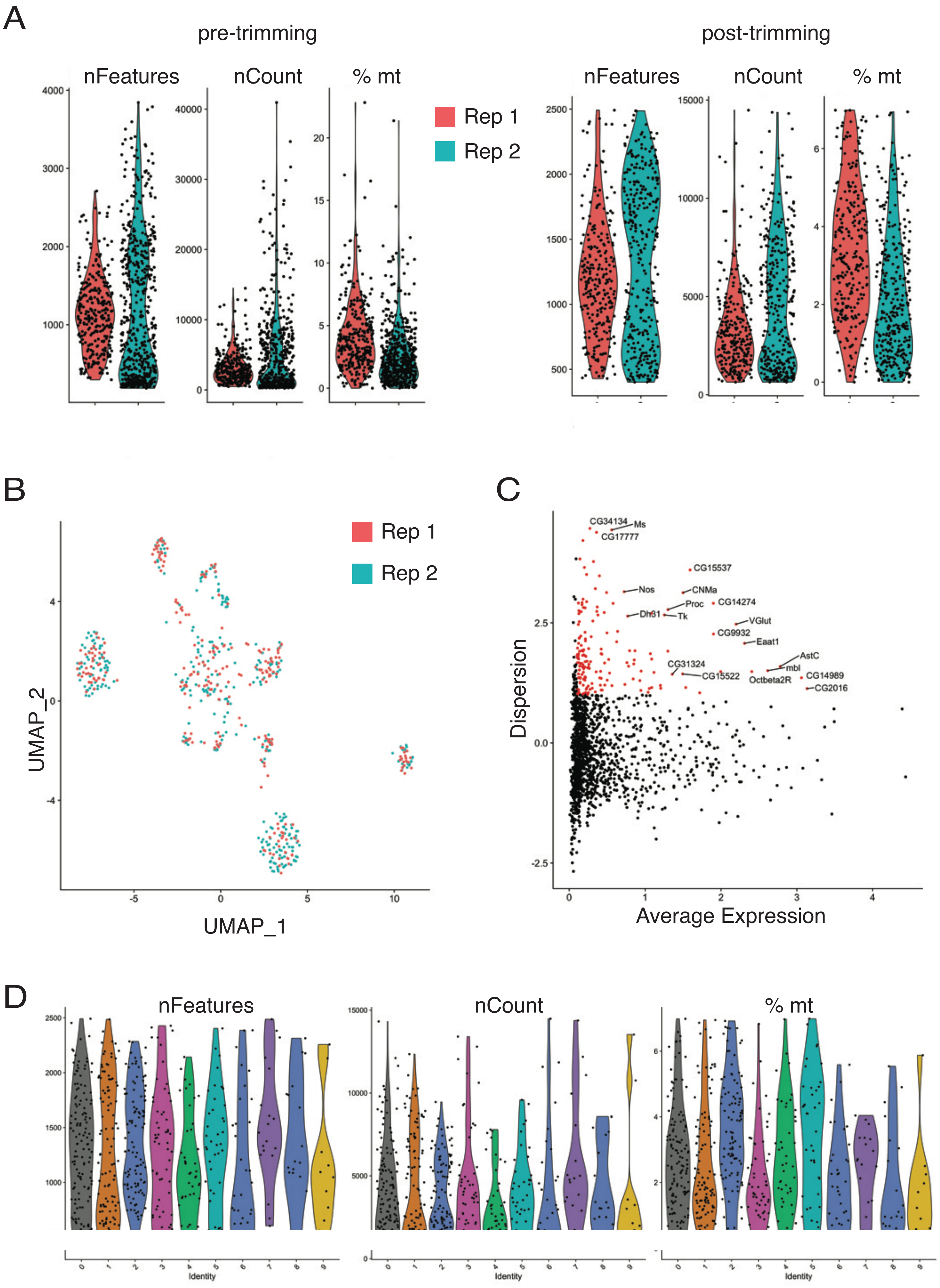

Fig. S3, Ehrlich et al.

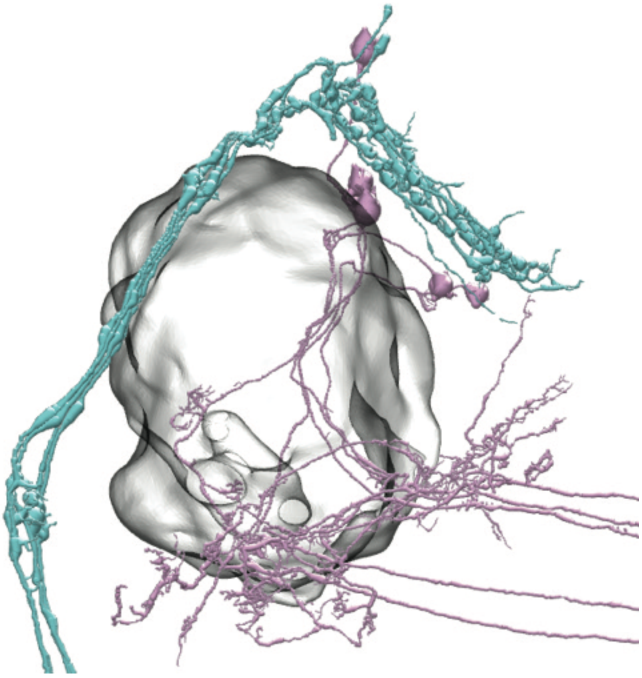

Fig. S4, Ehrlich et al.
